# miR156/SPL10 Modulates Lateral Root Development, Branching and Leaf Morphology in Arabidopsis by Silencing *AGAMOUS-LIKE 79*

**DOI:** 10.3389/fpls.2017.02226

**Published:** 2018-01-04

**Authors:** Ruimin Gao, Ying Wang, Margaret Y. Gruber, Abdelali Hannoufa

**Affiliations:** ^1^London Research and Development Center, Agriculture and Agri-Food Canada, London, ON, Canada; ^2^Saskatoon Research and Development Center, Agriculture and Agri-Food Canada, Saskatoon, SK, Canada

**Keywords:** Arabidopsis, *miR156*, *SPL10*, lateral root, *AGL79*, flowering time, leaf morphology

## Abstract

The developmental functions of miR156-SPL regulatory network have been extensively studied in Arabidopsis, but the downstream genes regulated by each SPL have not been well characterized. In this study, Next Generation Sequencing-based transcriptome analysis was performed on roots of wild type (WT) and miR156 overexpression (miR156OE) plants. One of the *SPL* genes, *SPL10*, which represses lateral root growth in Arabidopsis, was significantly downregulated in miR156OE plants. A transcription factor, *AGAMOUS-like MADS box protein 79* (*AGL79*), was also significantly downregulated in the miR156OE plants, but was upregulated in the *SPL10* overexpression (SPL10OE) Arabidopsis plants. In addition, SPL10 was found to bind to the core consensus SPL binding sequences in *AGL79* gene. Moreover, analyses of complementation lines revealed a linear relationship between *SPL10* and *AGL79* in regulating Arabidopsis plant development. In addition, it was observed that plant phenotypes are AGL79 dose-dependent, with higher expression causing narrow leaf shape, less number of leaves and early flowering time, whereas relatively lower AGL79 overexpression produce plants with more rosette leaves and more lateral branches. Our findings revealed direct binding of SPL10 to *AGL79* promoter, which further suggests a role for miR156/SPL10 module in plant lateral root growth by directly regulating *AGL79*.

## Introduction

MicroRNAs (miRNAs) are a class of non-coding RNAs with a length of 19–24 nucleotides that control gene expression at the posttranscriptional level (Bartel, 2004; Cuperus et al., 2011; Nozawa et al., 2012). Of all the miRNAs, miR156 is one of the most conserved in plants, where it regulates transition from the juvenile to the adult phase of vegetative development (Wu and Poethig, 2006; Chuck et al., 2007). MiR156, which is expressed mainly at the early stages of shoot development, targets and represses the expression of the gene family *SQUAMOSA PROMOTER BINDING PROTEIN-LIKE (SPL)* (Rhoades et al., 2002; Schwab et al., 2005; Wu and Poethig, 2006; Gandikota et al., 2007; Wu et al., 2009). The SPL protein family members possess a conserved squamosa promoter binding protein (SBP) domain of 76 amino acids (Yamasaki et al., 2004; Preston and Hileman, 2013) that binds to a consensus DNA element with a core GTAC sequence (Birkenbihl et al., 2005; Liang et al., 2008; Wei et al., 2012).

In Arabidopsis, 10 of 16 *SPL* genes are targeted by miR156 for silencing via transcript cleavage (Cardon et al., 1997; Rhoades et al., 2002; Schwab et al., 2006; Wu and Poethig, 2006; Xie et al., 2006). Based on the amino acid sequences of their conserved DNA binding domain, the 10 *SPLs* could be grouped into 5 clades; *SPL3/SPL4/SPL5, SPL9/SPL15, SPL2/SPL10/SPL11, SPL6, and SP13A/B* (Xie et al., 2006; Riese et al., 2007; Preston and Hileman, 2013). A genetic function study of each individual *SPL* gene in vegetative and reproductive phase development was also reported (Xu et al., 2016). Generally, based on this functional analysis, miR156-regulated *SPL* genes could be divided into three groups: (1) *SPL2, SPL9, SPL10, SPL11, SPL13*, and *SPL15* play crucial roles in both juvenile-to-adult vegetative transition and vegetative-to-reproductive transition. (2) *SPL3, SPL4*, and *SPL5* are involved in promoting the floral meristem identify transition. (3) *SPL6* is predicted to participate in regulating some physiological processes, but its exact function is still not fully understood (Xu et al., 2016).

Morphology of the plant root system is regulated by various factors, including numerous biotic and abiotic factors that make up the heterogeneous composition of the soil environment (Osmont et al., 2007) and soil matrix heterogeneity (Hodge, 2006), with the formation and growth of lateral roots being an important agronomic trait in plants (Yu et al., 2014). miR156-regulated *SPL* genes repressed the development of adventitious roots, for which production declined as plant growth progresses (Xu et al., 2016). Of all the known miR156 regulated *SPL* genes, only *SPL3, SPL9*, and *SPL10* participated in the repression of lateral root development, with *SPL10* playing a dominant role (Yu et al., 2015). In addition, *SPL10, SPL11* and *SPL2* redundantly controlled proper lateral organ development and shoot maturation in the reproductive phase, and ectopic expression of *SPL10* also altered leaf lamina shapes (Shikata et al., 2009). Expression of the *FRUITFULL* (*FUL*) gene increased with shoot maturation, while its expression was also reduced in the cauline leaves of *35S:SPL10SRDX* (a chimeric repressor) (Hiratsu et al., 2003) and increased in *35S:mSPL10/11/2* overexpression rosette leaves) (Shikata et al., 2009). These latter findings suggested that FUL may function in shoot maturation under the control of SPL proteins. In the leaf tissue, SPL2 controlled floral organ development and plant fertility by activating *AS2* (Wang et al., 2016).

MADS-box proteins are a family of transcription factors that are defined by their primary sequences, which encompass a conserved MADS-box motif; a 56-amino-acid region within the DNA-binding domain (Shore and Sharrocks, 1995). The majority of MADS-box proteins bind similar DNA elements with the consensus sequence CC(A/T)_6_GG, and several MADS-box proteins interact with other transcription factors to form multi-component regulatory complexes (Shore and Sharrocks, 1995). In plants, the MADS-box proteins are crucial for floral organ development and flowering time (Saedler and Huijser, 1993; Ma, 1994; Weigel and Meyerowitz, 1994). Specifically, SEPALLATA (SEP)-MADS-box subfamily factors are required for floral organ and meristem identity (Zahn et al., 2005). Another MADS box gene FLORAL BINDING PROTEIN 2 *(FBP2)* is required for *SEP* function in Petunia, and *FBP2* plays a similar role to that of *SEP3* in Arabidopsis (Ferrario et al., 2003). *LEAFY* (*LFY*) and *APETALA1 (AP1)* promote floral development not only by positively regulating genes activated in flower development, but also by repressing *AGAMOUS-LIKE* (*AGL24*), a promoter of inflorescence fate (Yu et al., 2004). These findings suggest that known functions of MADS-box proteins are mainly related to floral development.

Although *SPL* genes have been extensively studied in Arabidopsis aerial tissues, the regulatory pathways involving miR156, SPL and downstream SPL-regulated genes have not been thoroughly investigated and characterized in root tissues. To further study the underlying mechanisms of miR156-SPL10 network in Arabidopsis, we carried out RNA-Seq based transcriptome analysis on the root tissue of WT and miR156OE plants, to identify and characterize potential downstream genes that are downregulated by SPL10. The analysis provided an insight into the role of miR156-SPL10 network in regulating lateral root development and vegetative branching.

## Methods

### Plant materials, plasmid construction and generation of transgenic arabidopsis

All of the Arabidopsis stocks used in this study were developed in a Columbia (Col) genetic background. The mutant plants *spl2* (SALK_022235), *spl10* (SALK_122018) and *spl11* (SALK_112209), SPL10 overexpression lines (6mSPL10 and pSPL10-SPL10-GFP) (Nodine and Bartel, 2010), MIM156 seeds (Franco-Zorrilla et al., 2007) were obtained from the Arabidopsis Biological Resource Center (Ohio State University, Columbus, OH). Seeds of the *35S:miR156* were kindly provided by Dr. Detlef Weifel (Wang et al., 2008, 2009). All the Arabidopsis seeds were incubated at 4°C for 3 days in the dark for stratification, and then transferred to a growth room with long day conditions (16 h light, 8 h dark) and set at 23°C, 70% humidity, and a light intensity of 130–150 μmol/m^2^/s. Plasmid constructs were transformed individually into Arabidopsis ecotype Col-0 using the floral dip method (Zhang et al., 2006).

### Global gene expression analysis by NGS-based transcriptome analysis

RNA was extracted from the roots of both WT and 35S:miR156 Arabidopsis plants that were at the 20-day post germination stage. Four biological replicates (independent RNA preparations) were used for each genotype. NGS of the root RNA was performed by PlantBiosis (University of Lethbridge, Canada) under a fee-for-service contract. Using the *Arabidopsis* genome (TAIR10, http://www.arabidopsis.org/) as a reference, differential gene expression analysis was carried out based on published protocols (Trapnell et al., 2012). Briefly, raw sequencing data were first evaluated with the FastQC program. All filtered and properly paired reads were then mapped to the *Arabidopsis* genome using TopHat. The fragment alignments generated by TopHat were then used as input files to be further analyzed through the recommended Cufflinks packages to detect the differentially expressed genes between WT and 35S:miR156 Arabidopsis plants.

### Extraction of total RNA and qRT-PCR

Plant tissues were collected at specific time points as indicated in results for gene expression analysis. Total RNA was extracted using TRIzol reagent (Invitrogen) and 1 μg was used to generate cDNAs through reverse transcription, using oligo(dT)_15_ or gene specific reverse primers with a SuperScript® III Reverse Transcriptase kit (Invitrogen™. Expression levels of the selected transcripts were analyzed via qRT-PCR in a total volume of 10 μl and carried out in a 96-well plate on the CX96™ Real-Time PCR Detection System (Bio-Rad, California, United States). Each reaction consisted of 2 μl of cDNA template, 0.4 μl each of both gene-specific forward and reverse primers (10 μM) (Supplementary Table 2), and topped up to 10 μl with water. *CBP20* and *Tubulin* genes were used as internal controls for all qRT-PCR in Arabidopsis (Supplementary Table 2). Each test consisted of three biological sample repeats and each biological sample contained two technical replicates. Finally, transcript levels of the respective genes were analyzed using a relative quantification 2^−ΔCt^ method (Livak and Schmittgen, 2001).

### Analysis of protein-DNA interaction by ChIP-qPCR

Leaves from WT and SPL10-GFP transgenic Arabidopsis plants were used as materials for ChIP assays, which were performed according to a previously described protocol using the Chromatin Immunoprecipitation Assay kit (Lot:2382621, Millipore, Billerica, MS, United States) (Gendrel et al., 2005). Briefly, nuclei were isolated from leaves that were cross-linked with 1% formaldehyde under vacuum for 20–30 min and ground in liquid nitrogen. The chromatin solution was then sonicated 3 × 15 s into 500–1,000 bp fragments using a Sonic Dismembrator (Fisher Scientific, Hampton, New Hampshire, United States) set at power 3. Chromatin complexes were incubated with an anti-GFP antibody (Abcam, Cambridge, United Kingdom), and immune complexes were precipitated using Protein A beads. The precipitated DNA was purified and dissolved in water for further qPCR analysis using primers q_n_AtAGL79 as listed in Supplementary Table 2. SPL10 occupancy on *AGL79* was estimated by comparing the percentage of input (%input) in *pSPL10-SPL10-GFP* and WT plants (Yamaguchi et al., 2009). The consensus sequence “GTAC” was identified as the core binding motif of SPL proteins (Klein et al., 1996; Birkenbihl et al., 2005). Primers flanking the SPL10 binding core motif GTAC in the promoter region of *AGL79* were used to test for SPL10 occupancy. A DNA fragment containing a SBP binding consensus was amplified from an *EIF4A1* gene (Shuai et al., 2002) to serve as a negative control. All the primers used for ChIP-qPCR are listed in Supplementary Table 2.

### Western blot analysis and confocal microscope analyses

Fresh Arabidopsis leaves (0.1 g) were homogenized in 0.2 ml of protein extraction buffer (0.125 mM Tris, pH6.8, 4% w/v SDS, 18% glycerol, 0.024% w/v bromophenol-blue, 1.43 M β-mercaptoethanol, 0.2% protease inhibitor). After boiling for 10 min, the insoluble fraction was removed by centrifugation, and the supernatant (denatured protein) was separated on a 12% SDS PAGE gel and transferred onto a nitrocellulose membrane, followed by incubation with primary anti-GFP antibody (Abcam, ab290, Cambridge, MA, USA) and secondary goat anti-rabbit IgG HRP (Abcam) antibody. The membrane was developed with Pierce ECL Western Blotting Substrate (Thermo Fisher Scientific, Waltham, MA, USA). The expression of SPL10-GFP fusion protein was also investigated using a Biological Confocal Laser Scanning Microscope FV10-ASW, and the emission wavelength for GFP and DAPI channels are 488 nm and 405 nm, respectively (OLYMPUS, Tokyo, Japan).

## Results

### Overexpression of SPL10 reduces number and length of roots

**Figure 1:**
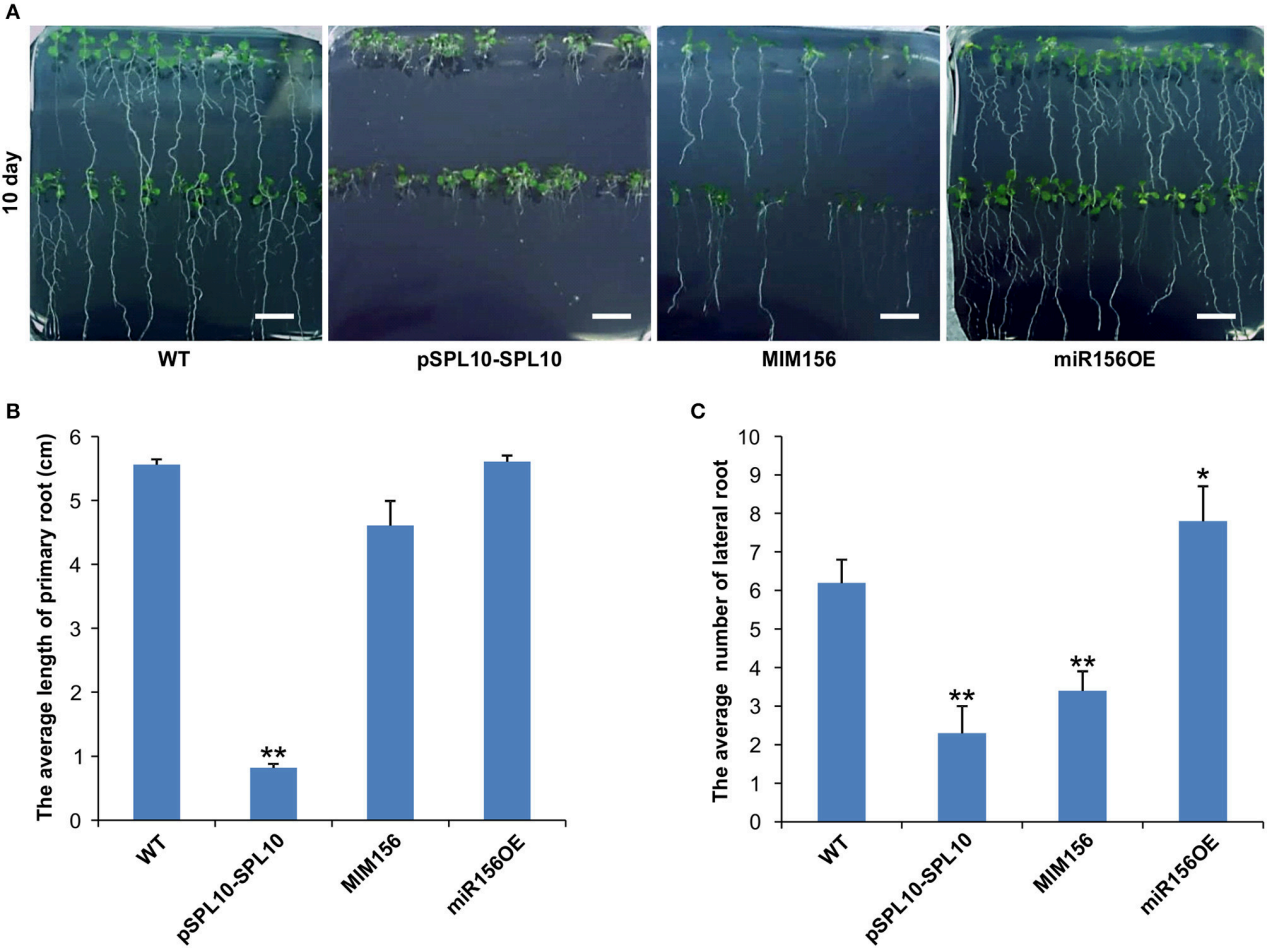
Effect of miR156 and SPL10 on root length. **(A)** Root morphology comparisons in WT, pSPL10-SPL10, MIM156 and miR156OE plants at 10 days after seed germination (bar = 1.2 cm). **(B)** The average length of the primary root and **(C)** the average number of lateral root among different tested genotypes. The primary root length and lateral root branch number were investigated in three independent experiments and each experiment consisted of three plates for each genotype (each plate consisted of approximately 20 plants). ^**^ and ^*^ represent significant differences relative to wild type using *t*-test at *p* < 0.01 and *p* < 0.05, respectively.

*SPL10* was reported earlier to play a dominant role in repressing lateral root development (Yu et al., 2015), so we investigated the root phenotypes in WT, pSPL10-SPL10-GFP (SPL10 overexpression under native promoter), miR156OE (miR156 overexpression) and MIM156 (miR156 repression) plants. As early as 10 days after seed germination, some differences could be observed among different Arabidopsis lines. Compared to WT plants, roots of pSPL10-SPL10-GFP plants were shorter with no obvious primary roots (Figures 1A,B), and have fewer lateral root branches (Figures 1A,C). MIM156 plants (where miR156 gene transcripts were suppressed) also had less lateral roots (Figures 1A,C). In contrast, the miR156OE plants showed relatively more lateral roots compared to WT control (Figures 1A,C). These results showed that the expression level of *SPL10* is negatively correlated to root development in Arabidopsis.

### Analysis of root transcriptomes in WT and miR156OE plants

**Figure 2:**
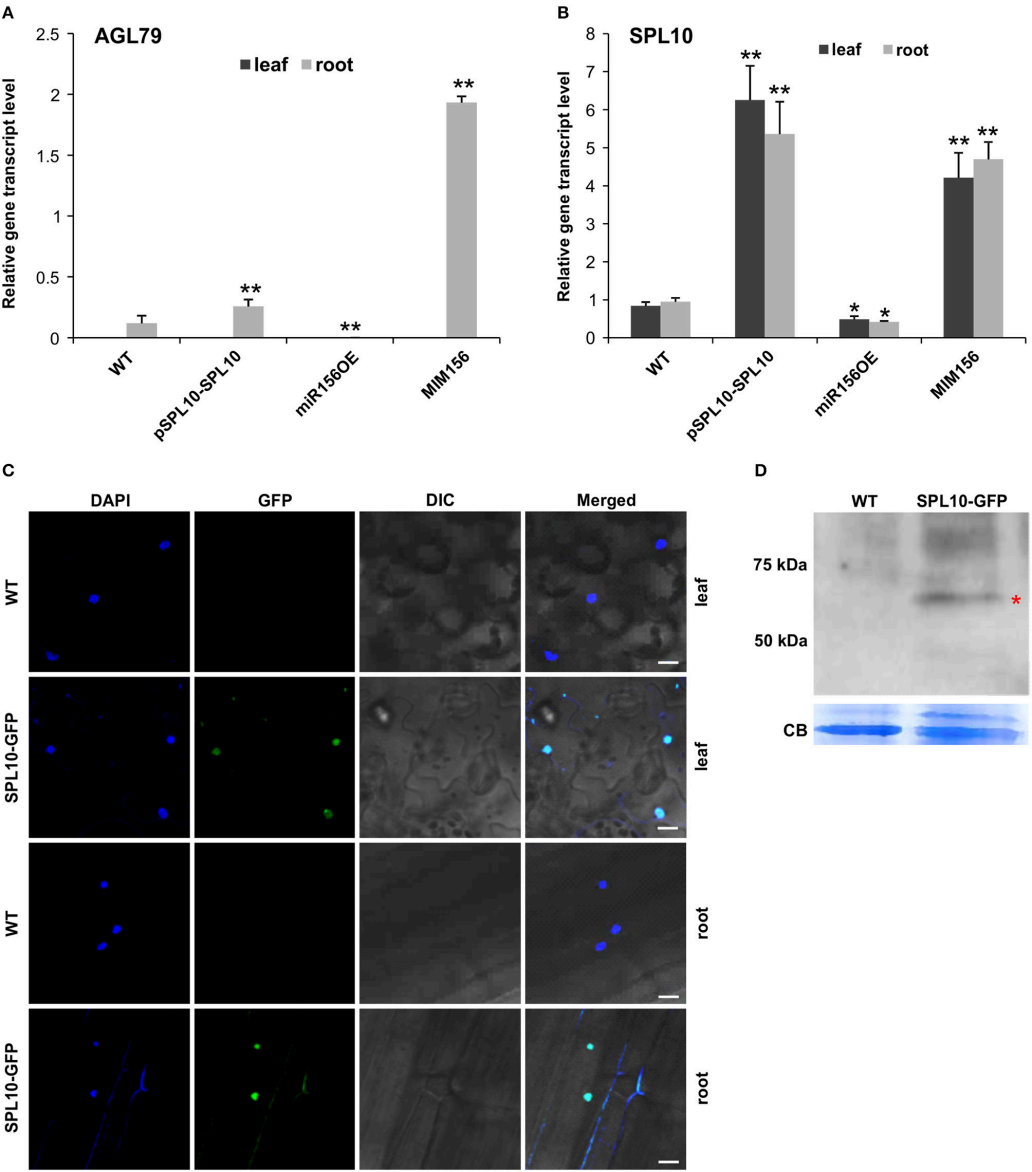
Detection of SPL10-GFP fusion protein from pSPL10-SPL10-GFP transgenic plants. Gene transcript level analysis of **(A)** *AGL79* and **(B)** *SPL10* in different genotypes (WT, pSPL10-SPL10, miR156OE and MIM156) of Arabidopsis. SPL10-GFP fusion protein was detected using both **(C)** confocal microscope (bar = 2.5 μm) and **(D)** western blot analysis with GFP as primary antibody. DIC: differential interference contrast; CB: Coomassie Blue Staining as loading control. ^**^ and ^*^ represent significant differences relative to wild type using *t*-test at *p* < 0.01 and *p* < 0.05, respectively.

In order to further identify genes that are involved in the miR156-SPL regulatory network in Arabidopsis, Next Generation Sequencing (NGS)-based transcriptome analysis was carried out on the root tissues of WT and miR156OE Arabidopsis plants. This analysis revealed a range of differentially expressed genes (DEG) between WT and miR156OE roots (Supplementary Table 1). Among all 10 miR156-targeted *SPL* genes, only *SPL10* and its homolog *SPL2* were significantly downregulated. Furthermore, a root gene encoding an uncharacterized transcription factor, *AGL79* (AT3G30260), was also significantly downregulated with the most prominent fold change (−5.59). Further expression analysis of *AGL79* revealed that it was nearly undetectable in leaf tissues of all Arabidopsis lines (WT, pSPL10-SPL10, miR156OE, and MIM156) (Figure 2A). This low leaf expression of *AGL79* is consistent with previous reports (Parenicova et al., 2003).

*AGL79* gene expression was initially characterized by us in several different Arabidopsis genotypes (WT, pSPL10-SPL10, miR156OE, and MIM156). In roots, *AGL79* levels were the highest in MIM156, followed by lower expression levels in pSPL10-SPL10-GFP and even lower levels in WT and miR156OE (Figure 2A). The transcript level of *SPL10* was also investigated in the above-mentioned genotypes. *SPL10* transcript was detected in both the leaf and root tissues (Figure 2B), and was highly expressed in both tissues of pSPL10-SPL10 and MIM156 plants, with much lower transcript levels in WT and miR156OE (Figure 2B). This expression trend is somewhat similar to that of *AGL79* (Figure 2A). The correlation between expression levels of *SPL10* and *AGL79* suggests that *AGL79* may be regulated by SPL10 through the miR156-SPL regulatory pathway.

### SPL10 directly binds to the *AGL79* promoter

As the afore-mentioned expression patterns suggested that *AGL79* might be regulated by SPL10, further characterization was carried out using ChIP-qPCR to determine if *AGL79* is a direct target of SPL10. For that, we characterized transgenic plants expressing the SPL10-GFP fusion protein (pSPL10-SPL10-GFP). Since SPL10 is a known transcription factor, we confirmed its nuclear localization in both the leaf and root tissues using confocal microscopy (Figure 2C). In addition, the SPL10-GFP fusion protein was also detected using western blot analysis (Figure 2D).

**Figure 3:**
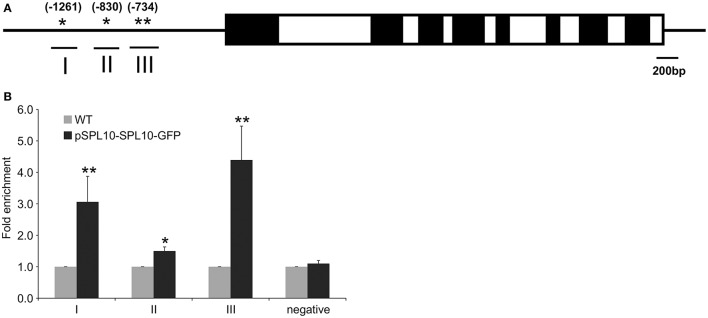
Detection of SPL10 binding AGL79 by CHIP-qPCR. **(A)** Schematic representation of the promoter region of AGL79; asterisks indicate the locations of putative SPL binding sites in the AGL79 promoter, and numbers in brackets indicate the relative position of binding sites to the translation start codon of AGL79. Roman numerals indicate the sites were tested by qPCR. **(B)** ChIP-qPCR enrichment of putative SPL binding sites I, II, III and negative control (–ve) EIF4A1 relative to WT (set at 1). Each ChIP-qPCR histogram indicates the mean ± standard error of the results of four biological replicates. Enrichment values were normalized to DNA input.

The upstream promoter region (2000 bp) of Arabidopsis *AGL79* revealed 4 core GTAC sequences that are distributed in three regions (I, II, and III), with all three regions possessing the typical NNGTACR SPL binding consensus (where *N* = any nucleotide, *R* = A or G) (Figure 3A, Supplementary Document 1). Strong binding capacity of SPL10 to regions I, II and III was detected by ChIP-qPCR in the pSPL10-SPL10-GFP transgenic Arabidopsis plants (Figures 3B–D). Compared to the WT control, occupancy in these three regions was substantially higher than that in the negative control eukaryotic translation initiation factor 4A1 (EIF4A1) (Figure 3E). Of the three putative SPL binding regions, region III showed a higher binding capacity (Figure 3D). These results show that the SPL10 protein could bind to multiple regions in the *AGL79* promoter. Therefore, AGL79 appears to be regulated through the miR156-SPL network to affect plant development in Arabidopsis.

### Phenotypic effects of *AtAGL79* misexpression in arabidopsis

**Figure 4:**
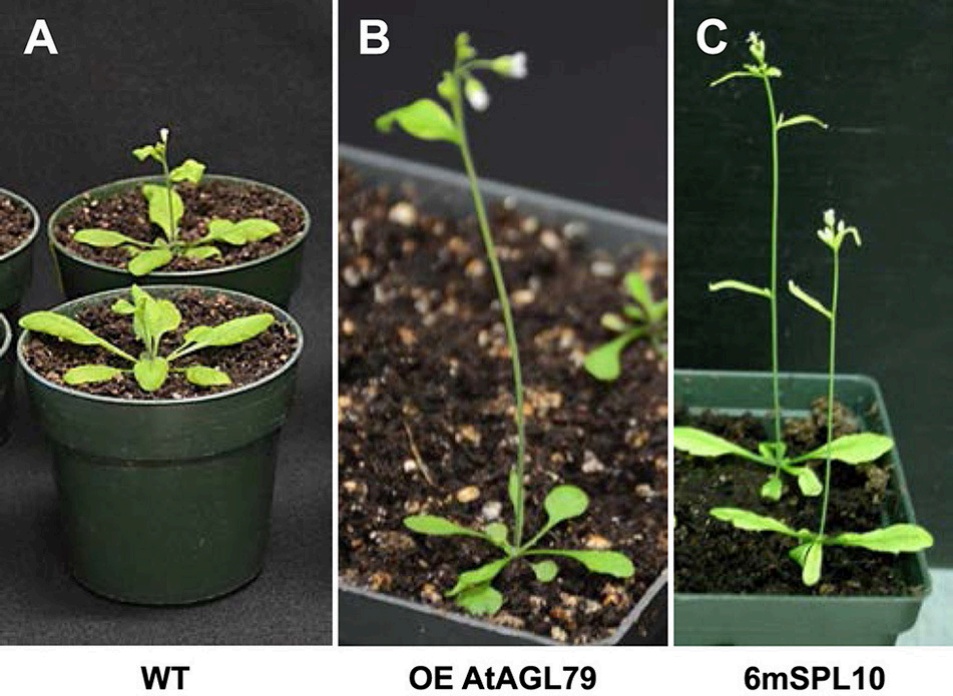
Phenotypic characterization of AGL79 misexpression Arabidopsis plants. All the used plants were grown at the same time and conditions, and the comparisons were carried out when WT reached the bolting stager. **(A)** WT plants. **(B)** Arabidopsis plants with highest *AGL79* gene over expression (OE). **(C)** Phenotypic display of *SPL10* overexpression line (6mSPL10) (bar = 1.1 cm).

To further investigate the role of *AGL79* in Arabidopsis development, we generated transgenic plants with either enhanced or silenced expression of *AGL79*. AGL79 overexpression Arabidopsis plants (Group 1, see next paragraph), on the other hand, had fewer and smaller rosette leaves, as well as earlier flowering time compared to WT plants at the same developmental stage [Figure 4B, 5B (WT and Group 1)]. SPL10 overexpression plants (6mSPL10) also showed a phenotype similar to that of AGL79OE (Group 1) plants (fewer and smaller rosette leaves) (Figure 4C, Supplementary Figure 2). The phenotypical similarities between AGL79OE and 6mSPL10 plants suggest a potential linear regulatory relationship between *AGL79* and *SPL10*. CRISPR-Cas9 was used to generate mutations in AGL79. Mutated plants were analyzed by Sanger sequencing, which detected mutations or deletions within the 20 bp sgRNA2 sequence regions (Supplementary Figure 1B), resulting in reduced gene expression (Supplementary Figure 1C). A phenotypic comparison between the four lines was carried out when the WT plants reached the bolting stage. Compared to WT (Figure 4A), CRISPR-Cas9-*AGL79* mutant plants had more lateral shoot branches (Figure 4D, Supplementary Figure 1A).

**Figure 5:**
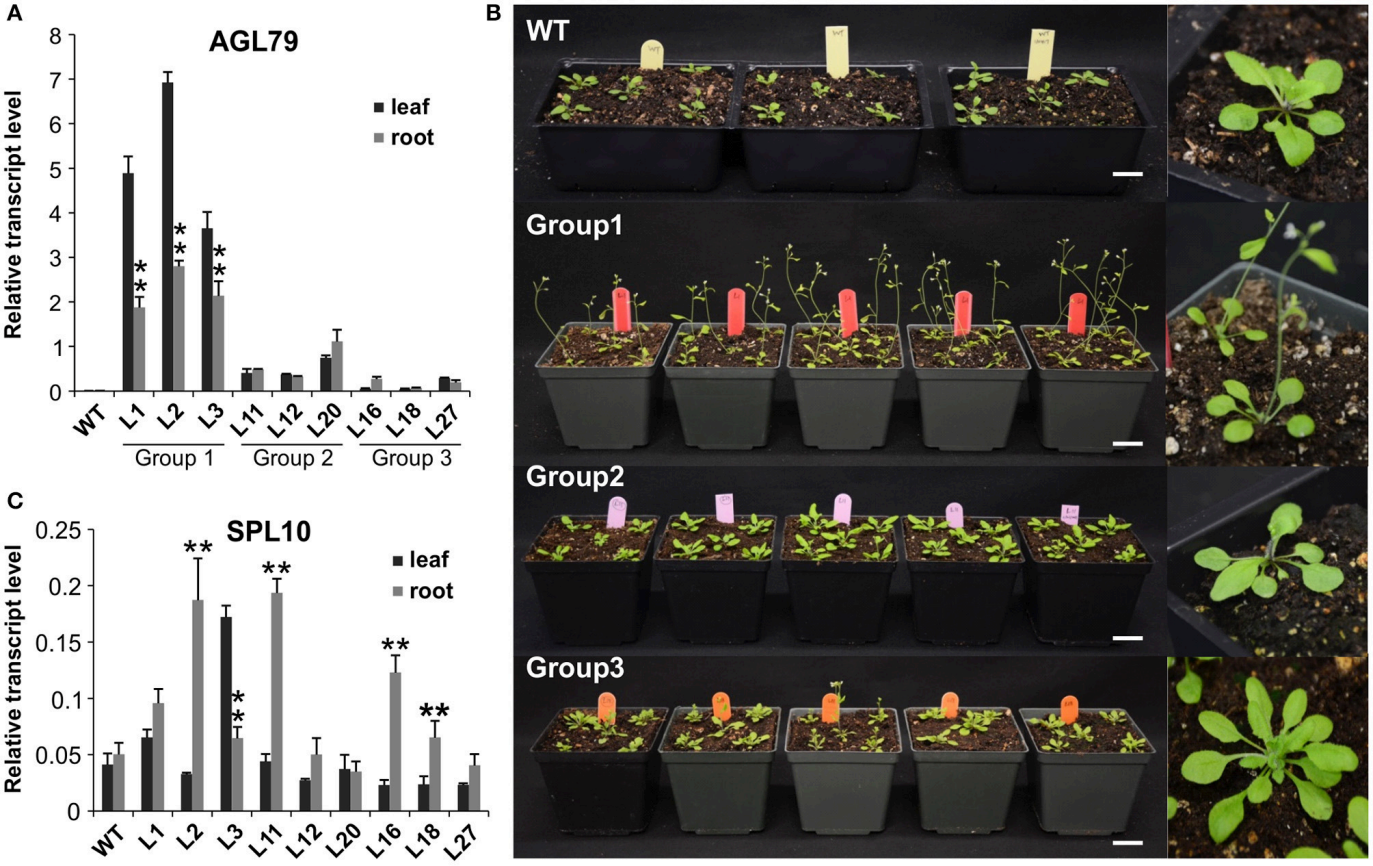
Comparison of Arabidopsis phenotypes with different *AGL79* expression levels. **(A)** *AGL79* gene expression in different groups of transgenic Arabidopsis plants. **(B)** Phenotype comparison of WT and different groups of AGL79 overexpression plants (bar = 3.5 cm). **(C)** Transcript levels of *SPL10* gene in different groups of *AGL79* overexpression plants. ^**^ represents significant downregulation in the root tissue relative to leaf tissue using *t*-test at *p* < 0.05.

**Figure 6:**
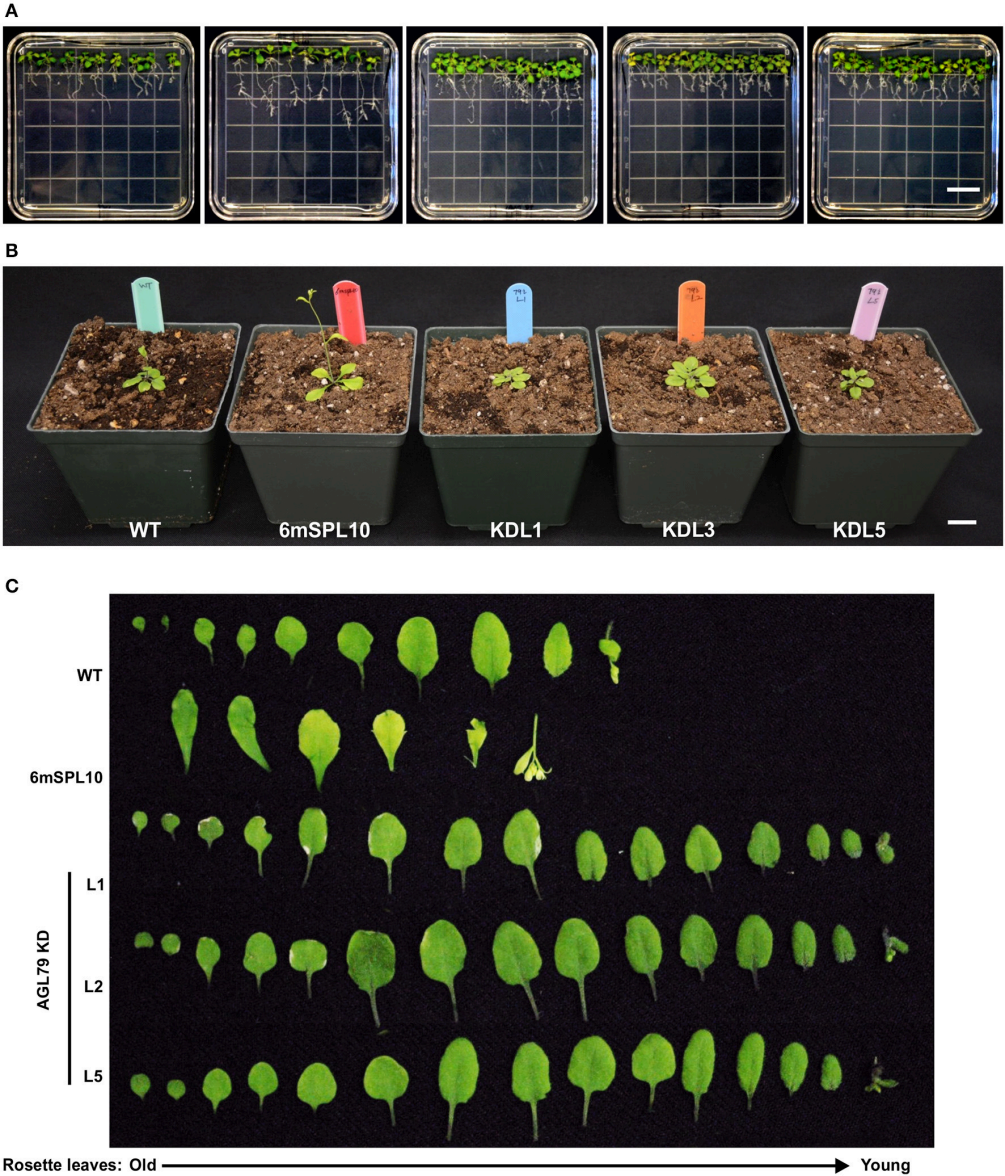
Morphological characterization of Arabidopsis plants with mutated *AGL79*. **(A)** Root morphology of AtAGL79 KD mutant at 14 days post germination. **(B,C)** Vegetative growth comparisons between WT, 6mSPL10 and three lines of AGL79KD mutant. Bar = 1.6 cm. Rosette leaves are shown from old (left) to young (right).

**Figure 7:**
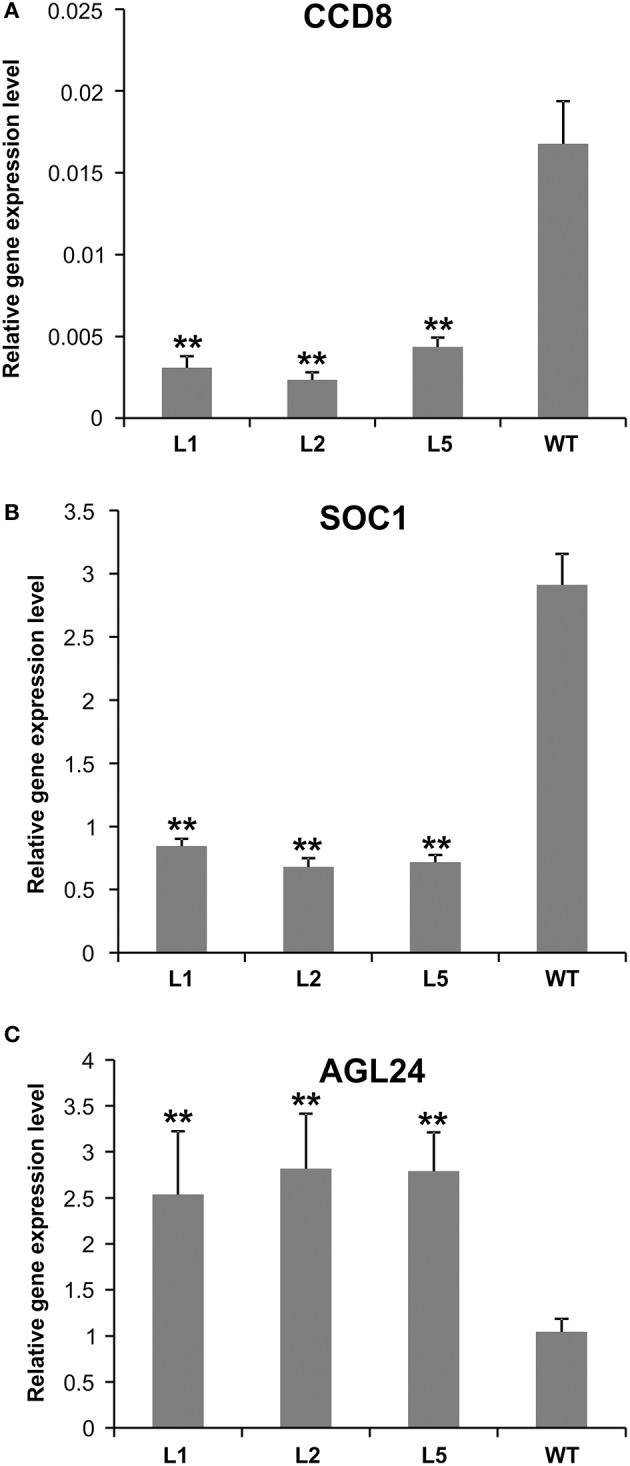
Expression analysis of flowering-related genes in AGL79 KD mutant. **(A)** *CCD8*, **(B)** *SOC1*, **(C)** *AGL24*. ^**^ and ^*^ represent significant differences relative to wild type using *t*-test at *p* < 0.01 and *p* < 0.05, respectively.

### Characterization of *AGL79* overexpression plants

To investigate the role of *AGL79* in Arabidopsis development, we generated transgenic plants with enhanced expression of *AGL79*. Compared to WT, the highest *AGL79* overexpression plants flowered early and had fewer and smaller rosette leaves [Figures 4B, 5B (WT and Group 1)] much like the SPL10 overexpression plants (6mSLP10) (Figure 4C, Supplementary Figure 1). Transgenic Arabidopsis plants harboring the *AGL79* overexpression construct were divided into three groups depending on AGL79 expression. Group 1 (lines L1, L2, and L3) had the highest *AGL79* transcript levels in both the leaf and root tissues (Figure 5A), with lower expression in roots relative to leaves. Group 2 (lines L11, L12, and L20) had intermediate *AGL79* expression, with variable expression levels between leaf and root (Figure 5A). Group 3 (lines L16, L18, and L27) displayed the lowest *AGL79* gene transcripts, and there were no obvious differences in *AGL79* transcript levels between the leaf and root (Figure 5A). Different phenotypes could be observed in these AGL79 overexpression plants depending on AGL79 expression levels (Figure 5B). Compared to WT (3 weeks after seed germination), Group 1 plants displayed fewer rosette leaves and early flowering time (Figure 5B). Group 2 plants displayed a phenotype similar to WT (Figure 5B). Group 3 plants showed more lateral shoot branches and a higher number of rosette leaves, as well as a significant delay in flowering (Figure 5B). In addition, the transcript level of *SPL10* gene was also investigated in both the leaves and roots of the above-mentioned plants. Although changes in *SPL10* expression could be detected in three groups of *AGL79*OE plants (Figure 5C), these changes did not follow any consistent trend, as found for *AGL79* (Figure 5C), suggesting that *AGL79* could be a downstream gene regulated by SPL10, and hence fluctuations in AGL79 expression would not affect the expression of the upstream *SPL10* gene.

### Regulatory relationship between *AGL79* and *SPL10*

**Figure 8:**
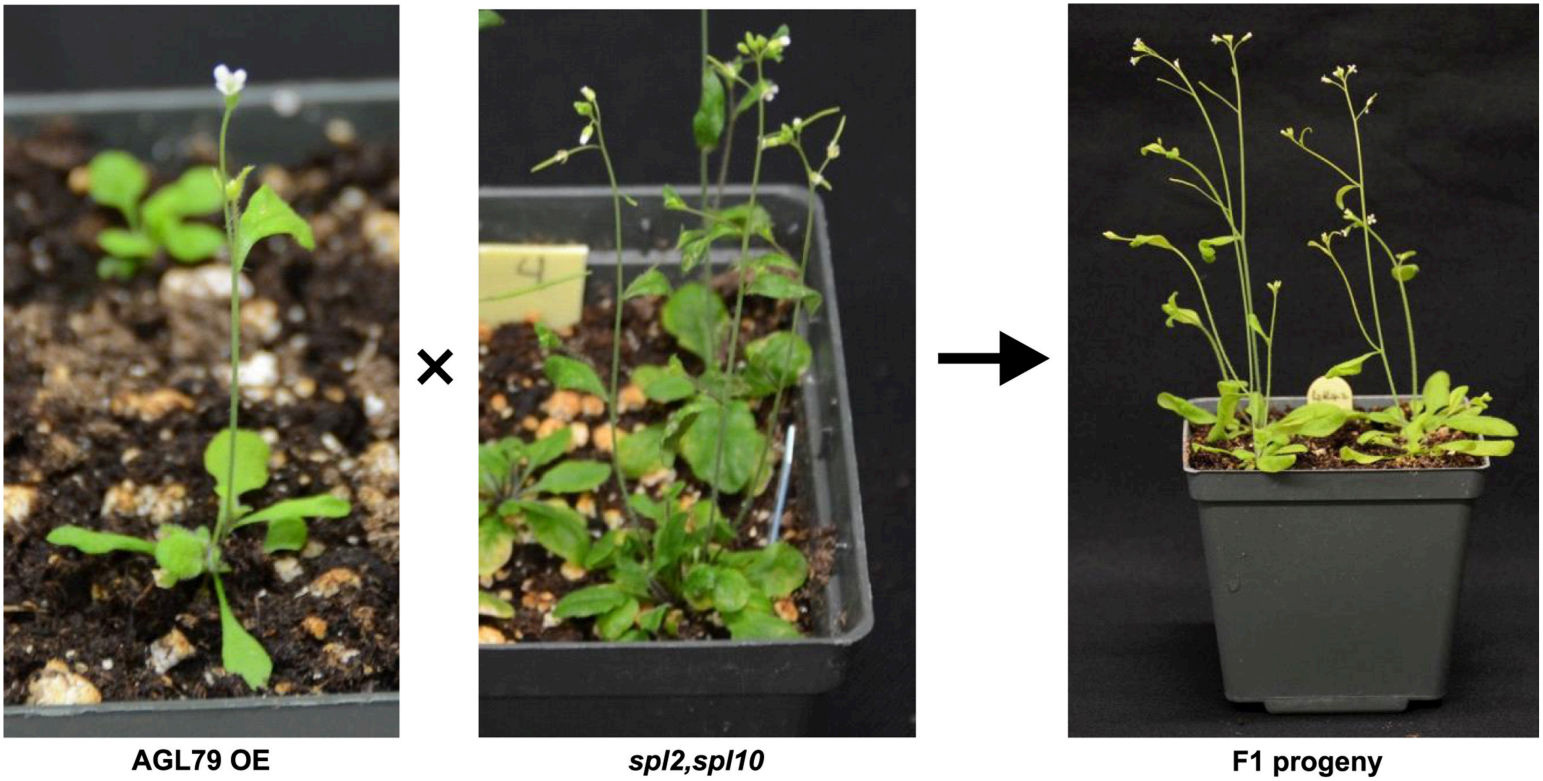
Complementary experiment to investigate relationship between *AGL79* and *SPL10*. WT phenotype was observed when crossing (*AGL79* overexpression plant and *spl2spl10* double mutant. Bar = 1.5cm.

As all the evidence derived from molecular and biological analysis (Figures 2A,B, 4C) revealed that AGL79 is likely regulated through the miR156-SPL pathway, we investigated whether a linear regulatory relationship exists between *SPL10* and *AGL79*. Crossing AGL79OE plants and *spl2spl10* double mutant produced F1 progeny showing WT-like phenotype (Figure 8B). The selected genotyping results of the double mutant (*spl2spl10*) and AGL79 OE plants are shown in Supplementary Figure 3. These results suggest a direct linear relationship between *AGL79* and *SPL10* genes.

## Discussion

In this study, NGS-based transcriptome analysis of root tissues revealed that both SPL10 and AGL79 were downregulated in miR156OE plants. Further analysis revealed that AGL79 is under the regulation of SPL10 and is involved in various aspects of Arabidopsis development, including branching of roots and shoots, as well as flowering.

The discovery that *AGL79* is regulated by *SPL10* may provide insight into how the latter regulates lateral root development in Arabidopsis (Yu et al., 2015). Currently lateral root formation in Arabidopsis is known to be regulated by two related *AUXIN RESPONSE FACTORS* (*ARF7* and *ARF19*) via direct activation of *LATERAL ORGAN BOUNDARIES DOMAIN* and *ASYMMETRIC LEAVES-LIKE* (*LBD/ASLs*) (Okushima et al., 2007). In addition, lateral root formation in Arabidopsis is also redundantly regulated by cytokinin biosynthesis genes *IPT3* and *IPT5* and all three cytokinin histidine kinase receptor genes (*AHK2, AHK3*, and *CRE1/AHK4*) (Chang et al., 2013). The plant hormones (auxin, cytokinins, gibberellins, abscisic acid, ethylene, jasmonic acid, strigolactones, brassinosteroids, and salicylic acid) also regulate normal root growth and mediate root morphological responses to abiotic stress (Chang et al., 2013). Morphological analysis of Arabidopsis plants with enhanced expression of *AGL79* revealed AGL79 to be involved in controlling shoot branching.

AGL79 also plays a role in regulating Arabidopsis leaf shape. High *AGL79* transcript levels altered leaf lamina shape in the AGL79OE plants, which was similar to the effect of *SPL10* overexpression (Figure 4D). During leaf development, *PIN1* and *KNOX1* are known to regulate leaf initiation, *HD-ZIPIII, KANADI*, and *YABBY* mediate leaf outgrowth, and *ANGUSTIFOLIA3* and *GROWTH-REGULATING FACTOR5* specify leaf expansion and maturation, while *APUM23* is also critical for determining leaf polarity (Dkhar and Pareek, 2014). It remains elusive whether *AGL79* and *SPL10* determine leaf shape in concert with the afore-mentioned plant leaf shape determination factors.

One interesting observation was that the phenotypes of AGL79 overexpression plants were *AGL79* dose-dependent. Generally, there were three major groups of phenotypes resulting from different levels of AGL79 expression: high (group 1), moderate (group 2) and low (group 3). The change in some phenotypes from group 1 to group 3 was gradual, such as with an increase in number of rosette leaves and shoot branches, but with decreasing days of flowering time. In group 1, we noted that *AGL79* gene transcript level was lower in the root tissue compared to that in the leaf tissue, which is contrary to WT where *AGL79* is mainly expressed in the root rather than the leaf. One possibility is that it is difficult to further overexpress *AGL79* gene in the roots, because the already high expression of the endogenous *AGL79* gene in this tissue (due to feedback regulation) prevents excessive overexpression of the transgene. Another possibility is that *AGL79* may play a dual role of acting simultaneously as an activator of leaf shape development in the leaf tissue and a repressor of lateral root development in the root tissue. The first identified WUSCHEL protein in Arabidopsis is a repressor of genes involved in the maintenance of stem cell population in shoot meristems and also an activator of *AGAMOUS*, which is involved in floral patterning (Ikeda et al., 2009). Arabidopsis *FILAMENTOUS FLOWER*, which controls lateral organ development, functions as an activator in regulating leaf patterning and a repressor to negatively regulate FIL-response genes (Bonaccorso et al., 2012). It is also possible that the observed *AGL79* overexpression phenotype might be due to dosage-dependent gene ectopic effect, as *AGL79* is barely detectable in WT leaf tissues.

In summary, our results suggest that the miR156/SPL10 regulatory pathway is involved in regulating plant lateral root growth by directly targeting and activating the expression of *AGL79*. By investigating the gain- of function of AGL79 transgenic plants, we also found AGL79 to be involved in regulating plant leaf shape, shoot branching, and flowering time. Further characterization of the *AGL79* gene in other plant species, especially in major crops, will determine how conserved AGL79 is in plants. It can also be tested in crop improvement efforts to enhance resilience and productivity.

## Author contributions

RG and AH. designed and managed the project; YW conduced RNA-Seq analysis. RG performed experiments and drafted the manuscript. MG and AH provided intellectual input and revised the manuscript.

### Conflict of interest statement

The authors declare that the research was conducted in the absence of any commercial or financial relationships that could be construed as a potential conflict of interest.

## Supplementary material

Supplementary Figure 1 AGL79 knockout mutant using CRISPR technique. **(A)** Vegetative morphology of WT and three lines of AGL79 CRISPR Plants (bar = 1.5 cm). **(B)** Genomic DNA sequence analysis of CRISPR-modified Arabidopsis plants. **(C)** Detecting *AGL79* transcript levels by qRT-PCR in WT and three lines of AGL79 CRISPR Plants.

Supplementary Figure 2 Phenotypes of a batch of 6mSPL10 plants (bar = 1.5 cm).

Supplementary Figure 3 Selected genotyping results from plants of **(A)** *spl2spl10* double mutant and **(B)** AGL79 OE.

Supplementary Table 1 Differentially expressed genes between WT and miR156OE Arabidopsis plant roots.

Supplementary Table 2 Primers used in this study.

Supplementary Document 1 The 2,000 bp upstream promoter region of AGL79 gene.

## References

[B1] Bartel D. P. (2004). MicroRNAs: genomics, biogenesis, mechanism, and function. Cell 116, 281–297. 10.1016/S0092-8674(04)00045-5 14744438

[B2] Birkenbihl R. P. Jach G. Saedler H. Huijser P. (2005). Functional dissection of the plant-specific SBP-domain: overlap of the DNA-binding and nuclear localization domains. J. Molecul. Biol. 352, 585–596. 10.1016/j.jmb.2005.07.013 16095614

[B3] Bonaccorso O. Lee J. E. Puah L. Scutt C. P. Golz J. F. (2012). FILAMENTOUS FLOWER controls lateral organ development by acting as both an activator and a repressor. BMC Plant Biol. 12:176. 10.1186/1471-2229-12-176 23025792 PMC3520853

[B4] Cardon G. H. Hohmann S. Nettesheim K. Saedler H. Huijser P. (1997). Functional analysis of the *Arabidopsis thaliana* SBP-box gene SPL3: a novel gene involved in the floral transition. Plant J. 12, 367–377. 10.1046/j.1365-313X.1997.12020367.x 9301089

[B5] Chang L. Ramireddy E. Schmulling T. (2013). Lateral root formation and growth of Arabidopsis is redundantly regulated by cytokinin metabolism and signalling genes. J. Exp. Bot. 64, 5021–5032. 10.1093/jxb/ert291 24023250 PMC3830484

[B6] Chuck G. Cigan A. M. Saeteurn K. Hake S. (2007). The heterochronic maize mutant Corngrass1 results from overexpression of a tandem microRNA. Nat. Genet. 39, 544–549. 10.1038/ng2001 17369828

[B7] Cuperus J. T. Fahlgren N. Carrington J. C. (2011). Evolution and functional diversification of MIRNA genes. Plant Cell 23, 431–442. 10.1105/tpc.110.082784 21317375 PMC3077775

[B8] Dkhar J. Pareek A. (2014). What determines a leaf's shape? Evodevo 5:47. 10.1186/2041-9139-5-47 25584185 PMC4290414

[B9] Ferrario S. Immink R. G. Shchennikova A. Busscher-Lange J. Angenent G. C. (2003). The MADS box gene FBP2 is required for SEPALLATA function in petunia. Plant Cell 15, 914–925. 10.1105/tpc.010280 12671087 PMC152338

[B10] Franco-Zorrilla J. M. Valli A. Todesco M. Mateos I. Puga M. I. Rubio-Somoza I.. (2007). Target mimicry provides a new mechanism for regulation of microRNA activity. Nat. Genet. 39, 1033–1037. 10.1038/ng2079 17643101

[B11] Gandikota M. Birkenbihl R. P. Hohmann S. Cardon G. H. Saedler H. Huijser P. (2007). The miRNA156/157 recognition element in the 3' UTR of the Arabidopsis SBP box gene SPL3 prevents early flowering by translational inhibition in seedlings. Plant J. 49, 683–693. 10.1111/j.1365-313X.2006.02983.x 17217458

[B12] Gendrel A. V. Lippman Z. Martienssen R. Colot V. (2005). Profiling histone modification patterns in plants using genomic tiling microarrays. Nat. Methods 2, 213–218. 10.1038/nmeth0305-213 16163802

[B13] Hiratsu K. Matsui K. Koyama T. Ohme-Takagi M. (2003). Dominant repression of target genes by chimeric repressors that include the EAR motif, a repression domain, in Arabidopsis. Plant J. 34, 733–739. 10.1046/j.1365-313X.2003.01759.x 12787253

[B14] Hodge A. (2006). Plastic plants and patchy soils. J. Exp. Bot. 57, 401–411. 10.1093/jxb/eri280 16172138

[B15] Ikeda M. Mitsuda N. Ohme-Takagi M. (2009). Arabidopsis WUSCHEL is a bifunctional transcription factor that acts as a repressor in stem cell regulation and as an activator in floral patterning. Plant Cell 21, 3493–3505. 10.1105/tpc.109.069997 19897670 PMC2798335

[B16] Klein J. Saedler H. Huijser P. (1996). A new family of DNA binding proteins includes putative transcriptional regulators of the *Antirrhinum majus* floral meristem identity gene SQUAMOSA. Mol. Gen. Genet. 250, 7–16. 8569690 10.1007/BF02191820

[B17] Liang X. Nazarenus T. J. Stone J. M. (2008). Identification of a consensus DNA-binding site for the *Arabidopsis thaliana* SBP domain transcription factor, AtSPL14, and binding kinetics by surface plasmon resonance. Biochemistry 47, 3645–3653. 10.1021/bi701431y 18302343

[B18] Livak K. J. Schmittgen T. D. (2001). Analysis of relative gene expression data using real-time quantitative PCR and the 2^−ΔΔ*C*_T_^ Method. Methods 25, 402–408. 10.1006/meth.2001.1262 11846609

[B19] Ma H. (1994). The unfolding drama of flower development: recent results from genetic and molecular analyses. Genes Dev. 8, 745–756. 10.1101/gad.8.7.745 7926764

[B20] Nodine M. D. Bartel D. P. (2010). MicroRNAs prevent precocious gene expression and enable pattern formation during plant embryogenesis. Genes Dev. 24, 2678–2692. 10.1101/gad.1986710 21123653 PMC2994041

[B21] Nozawa M. Miura S. Nei M. (2012). Origins and evolution of microRNA genes in plant species. Genome Biol. Evol. 4, 230–239. 10.1093/gbe/evs002 22223755 PMC3318440

[B22] Okushima Y. Fukaki H. Onoda M. Theologis A. Tasaka M. (2007). ARF7 and ARF19 regulate lateral root formation via direct activation of LBD/ASL genes in Arabidopsis. Plant Cell 19, 118–130. 10.1105/tpc.106.047761 17259263 PMC1820965

[B23] Osmont K. S. Sibout R. Hardtke C. S. (2007). Hidden branches: developments in root system architecture. Annu. Rev. Plant Biol. 58, 93–113. 10.1146/annurev.arplant.58.032806.104006 17177637

[B24] Parenicova L. De Folter S. Kieffer M. Horner D. S. Favalli C. Busscher J.. (2003). Molecular and phylogenetic analyses of the complete MADS-box transcription factor family in Arabidopsis: new openings to the MADS world. Plant Cell 15, 1538–1551. 10.1105/tpc.011544 12837945 PMC165399

[B25] Preston J. C. Hileman L. C. (2013). Functional evolution in the plant SQUAMOSA-PROMOTER BINDING PROTEIN-LIKE (SPL) gene family. Front. Plant Sci. 4:80. 10.3389/fpls.2013.00080 23577017 PMC3617394

[B26] Rhoades M. W. Reinhart B. J. Lim L. P. Burge C. B. Bartel B. Bartel D. P. (2002). Prediction of plant microRNA targets. Cell 110, 513–520. 10.1016/S0092-8674(02)00863-2 12202040

[B27] Riese M. Hohmann S. Saedler H. Munster T. Huijser P. (2007). Comparative analysis of the SBP-box gene families in P. patens and seed plants. Gene 401, 28–37. 10.1016/j.gene.2007.06.018 17689888

[B28] Saedler H. Huijser P. (1993). Molecular biology of flower development in *Antirrhinum majus* (snapdragon). Gene 135, 239–243. 10.1016/0378-1119(93)90071-A 8276263

[B29] Schwab R. Ossowski S. Riester M. Warthmann N. Weigel D. (2006). Highly specific gene silencing by artificial microRNAs in Arabidopsis. Plant Cell 18, 1121–1133. 10.1105/tpc.105.039834 16531494 PMC1456875

[B30] Schwab R. Palatnik J. F. Riester M. Schommer C. Schmid M. Weigel D. (2005). Specific effects of microRNAs on the plant transcriptome. Dev Cell 8, 517–527. 10.1016/j.devcel.2005.01.018 15809034

[B31] Shikata M. Koyama T. Mitsuda N. Ohme-Takagi M. (2009). Arabidopsis SBP-box genes SPL10, SPL11 and SPL2 control morphological change in association with shoot maturation in the reproductive phase. Plant Cell Physiol. 50, 2133–2145. 10.1093/pcp/pcp148 19880401

[B32] Shore P. Sharrocks A. D. (1995). The MADS-box family of transcription factors. Eur. J. Biochem. 229, 1–13. 10.1111/j.1432-1033.1995.tb20430.x 7744019

[B33] Shuai B. Reynaga-Pena C. G. Springer P. S. (2002). The lateral organ boundaries gene defines a novel, plant-specific gene family. Plant Physiol. 129, 747–761. 10.1104/pp.010926 12068116 PMC161698

[B34] Trapnell C. Roberts A. Goff L. Pertea G. Kim D. Kelley D. R.. (2012). Differential gene and transcript expression analysis of RNA-seq experiments with TopHat and Cufflinks. Nat. Protoc. 7, 562–578. 10.1038/nprot.2012.016 22383036 PMC3334321

[B35] Wang J. W. Czech B. Weigel D. (2009). miR156-regulated SPL transcription factors define an endogenous flowering pathway in *Arabidopsis thaliana*. Cell 138, 738–749. 10.1016/j.cell.2009.06.014 19703399

[B36] Wang J. W. Schwab R. Czech B. Mica E. Weigel D. (2008). Dual effects of miR156-targeted SPL genes and CYP78A5/KLUH on plastochron length and organ size in *Arabidopsis thaliana*. Plant Cell 20, 1231–1243. 10.1105/tpc.108.058180 18492871 PMC2438454

[B37] Wang Z. Wang Y. Kohalmi S. E. Amyot L. Hannoufa A. (2016). SQUAMOSA PROMOTER BINDING PROTEIN-LIKE 2 controls floral organ development and plant fertility by activating ASYMMETRIC LEAVES 2 in *Arabidopsis thaliana*. Plant Mol. Biol. 92, 661–674. 10.1007/s11103-016-0536-x 27605094

[B38] Wei S. Gruber M. Y. Yu B. Gao M. J. Khachatourians G. G. Hegedus D. D.. (2012). Arabidopsis mutant sk156 reveals complex regulation of SPL15 in a miR156-controlled gene network. BMC Plant Biol. 12:169. 10.1186/1471-2229-12-169 22989211 PMC3520712

[B39] Weigel D. Meyerowitz E. M. (1994). The ABCs of floral homeotic genes. Cell 78, 203–209. 10.1016/0092-8674(94)90291-7 7913881

[B40] Wu G. Poethig R. S. (2006). Temporal regulation of shoot development in *Arabidopsis thaliana* by miR156 and its target SPL3. Development 133, 3539–3547. 10.1242/dev.02521 16914499 PMC1610107

[B41] Wu G. Park M. Y. Conway S. R. Wang J. W. Weigel D. Poethig R. S. (2009). The sequential action of miR156 and miR172 regulates developmental timing in Arabidopsis. Cell 138, 750–759. 10.1016/j.cell.2009.06.031 19703400 PMC2732587

[B42] Xie K. Wu C. Xiong L. (2006). Genomic organization, differential expression, and interaction of SQUAMOSA promoter-binding-like transcription factors and microRNA156 in rice. Plant Physiol. 142, 280–293. 10.1104/pp.106.084475 16861571 PMC1557610

[B43] Xu M. Hu T. Zhao J. Park M. Y. Earley K. W. Wu G.. (2016). Developmental functions of miR156-regulated SQUAMOSA PROMOTER BINDING PROTEIN-LIKE (SPL) genes in *Arabidopsis thaliana*. PLoS Genet. 12:e1006263. 10.1371/journal.pgen.1006263 27541584 PMC4991793

[B44] Yamaguchi A. Wu M. F. Yang L. Wu G. Poethig R. S. Wagner D. (2009). The microRNA-regulated SBP-Box transcription factor SPL3 is a direct upstream activator of LEAFY, FRUITFULL, and APETALA1. Dev. Cell 17, 268–278. 10.1016/j.devcel.2009.06.007 19686687 PMC2908246

[B45] Yamasaki K. Kigawa T. Inoue M. Tateno M. Yamasaki T. Yabuki T.. (2004). A novel zinc-binding motif revealed by solution structures of DNA-binding domains of Arabidopsis SBP-family transcription factors. J. Mol. Biol. 337, 49–63. 10.1016/j.jmb.2004.01.015 15001351

[B46] Yu H. Ito T. Wellmer F. Meyerowitz E. M. (2004). Repression of AGAMOUS-LIKE 24 is a crucial step in promoting flower development. Nat. Genet. 36, 157–161. 10.1038/ng1286 14716314

[B47] Yu L. H. Miao Z. Q. Qi G. F. Wu J. Cai X. T. Mao J. L.. (2014). MADS-box transcription factor AGL21 regulates lateral root development and responds to multiple external and physiological signals. Mol. Plant 7, 1653–1669. 10.1093/mp/ssu088 25122697 PMC4228986

[B48] Yu N. Niu Q. W. Ng K. H. Chua N. H. (2015). The role of miR156/SPLs modules in Arabidopsis lateral root development. Plant J. 83, 673–685. 10.1111/tpj.12919 26096676

[B49] Zahn L. M. Kong H. Leebens-Mack J. H. Kim S. Soltis P. S. Landherr L. L.. (2005). The evolution of the SEPALLATA subfamily of MADS-box genes: a preangiosperm origin with multiple duplications throughout angiosperm history. Genetics 169, 2209–2223. 10.1534/genetics.104.037770 15687268 PMC1449606

[B50] Zhang X. Henriques R. Lin S. S. Niu Q. W. Chua N. H. (2006). Agrobacterium-mediated transformation of Arabidopsis thaliana using the floral dip method. Nat. Protoc. 1, 641–646. 10.1038/nprot.2006.97 17406292

