# Corrigendum: miR156/SPL10 Modulates Lateral Root Development, Branching and Leaf Morphology in Arabidopsis by Silencing *AGAMOUS-LIKE 79*

**DOI:** 10.3389/fpls.2019.00515

**Published:** 2019-05-09

**Authors:** Ruimin Gao, Ying Wang, Margaret Y. Gruber, Abdelali Hannoufa

**Affiliations:** ^1^London Research and Development Center, Agriculture and Agri-Food Canada, London, ON, Canada; ^2^Saskatoon Research and Development Center, Agriculture and Agri-Food Canada, Saskatoon, SK, Canada

**Keywords:** Arabidopsis, *miR156*, *SPL10*, lateral root, *AGL79*, flowering time, leaf morphology

In the original article, there was a mistake in the legend for Figure 4D, Figure 8A as published. There was an error in our CRISPR-generated AGL79KD mutant. In the **Results**, subsection **Characterization of the AGL79KD Arabidopsis Mutant**, we showed Arabidopsis knockdown mutants with a CRISPR-mutated AGL79. In performing follow up research we discovered that the plants had no point mutations as claimed in the original manuscript. As the researcher who carried out this experiment left the lab about 18 months ago, we reviewed the original sequence traces, and found that when reading the sequence in one direction (as the researcher probably did) it seemed to indicate point mutations, but when we sequenced in the reverse direction we could find no such point mutations in the AGL79 locus, and determined that this was indeed an error in reading the sequencing data. Figure legends that included this mutant have been modified to omit ALG79KD (Figures 4D and 8A).

**Figure 4:**
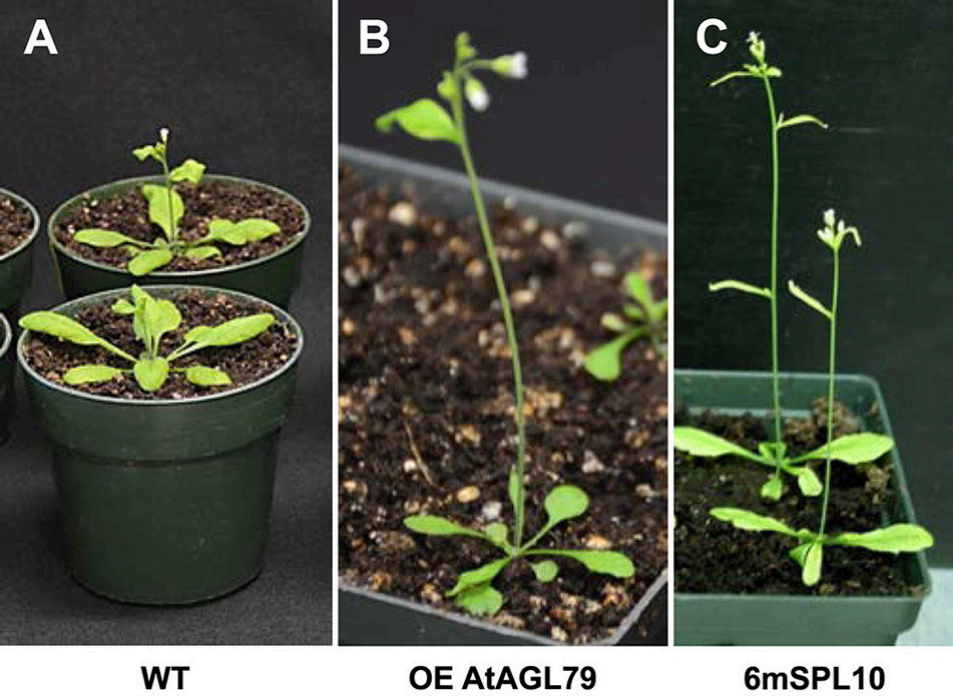
Phenotypic characterization of AGL79 misexpression Arabidopsis plants. All the used plants were grown at the same time and conditions, and the comparisons were carried out when WT reached the bolting stager. **(A)** WT plants. **(B)** Arabidopsis plants with highest *AGL79* gene over expression (OE). **(C)** Phenotypic display of *SPL10* overexpression line (6mSPL10) (bar = 1.1 cm).

**Figure 8:**
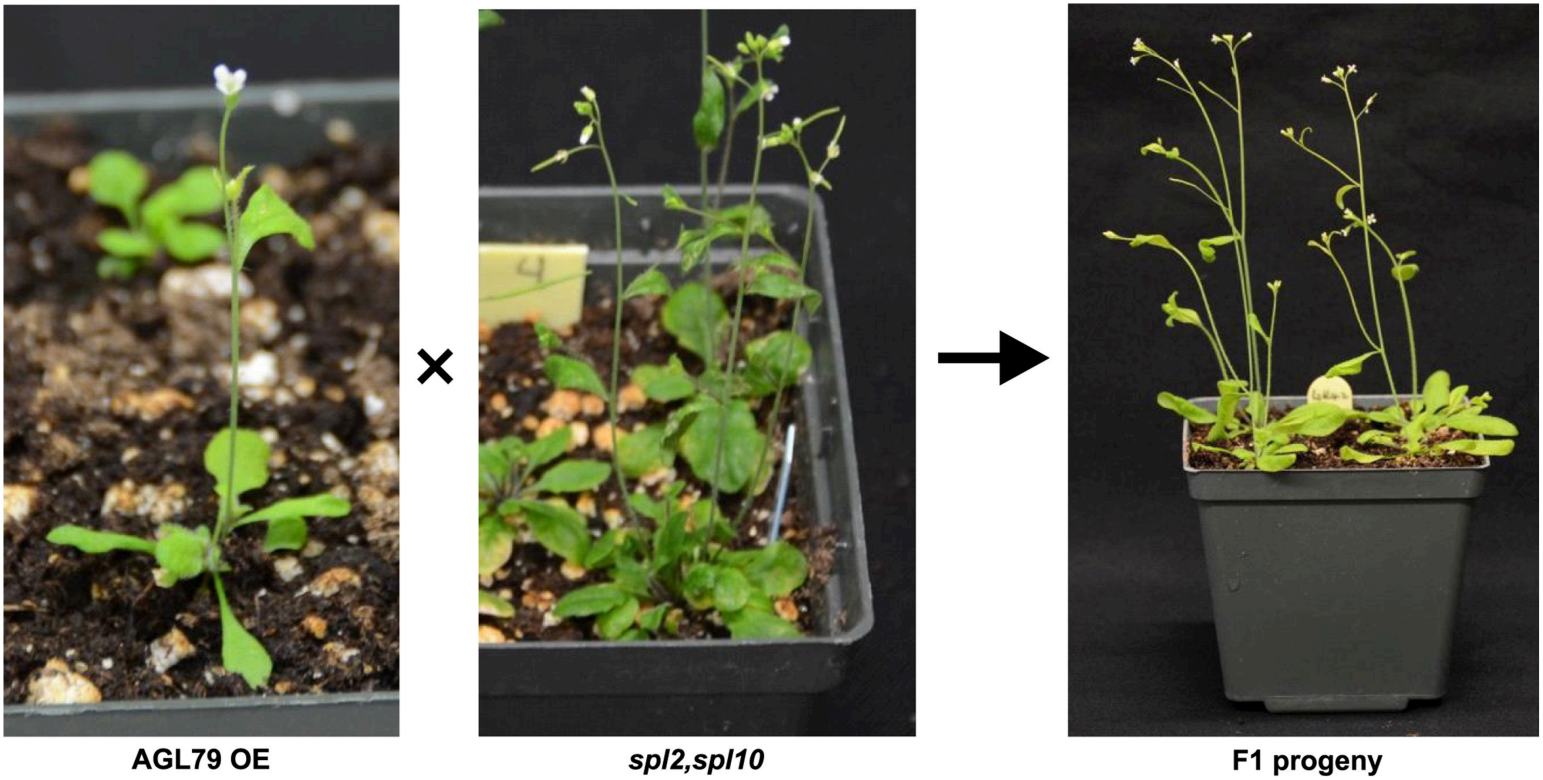
Complementary experiment to investigate relationship between *AGL79* and *SPL10*. WT phenotype was observed when crossing (*AGL79* overexpression plant and *spl2spl10* double mutant. Bar = 1.5cm.

Due to the same error detailed above, there was a mistake in Figures 6, 7, Supplementary Figure 1 and the Supplementary Material Document 2. Figures and their corresponding legends that were specific to AGL79KD have been removed (Figures 6, 7, Supplementary Figure 1, and Supplementary Material Document 2). Figures have also been renumbered to reflect these changes. In addition, primers related to the generation and validation of CRISPR plants have been removed from Supplementary Table 2.

Furthermore, in the original article, the following eight references, that are related to AGL79 KD mutant descriptions/discussions, have been removed.

1. An, H., Roussot, C., Suarez-Lopez, P., Corbesier, L., Vincent, C., Pineiro, M., et al. (2004). CONSTANS acts in the phloem to regulate a systemic signal that induces photoperiodic flowering of Arabidopsis. *Development* 131, 3615–3626. doi: 10.1242/dev.01231
2. Bennett, T., Sieberer, T., Willett, B., Booker, J., Luschnig, C., and Leyser, O. (2006). The Arabidopsis MAX pathway controls shoot branching by regulating auxin transport. *Curr. Biol*. 16, 553–563. doi: 10.1016/j.cub.2006.01.058
3. Lee, J. H., Yoo, S. J., Park, S. H., Hwang, I., Lee, J. S., and Ahn, J. H. (2007). Role of SVP in the control of flowering time by ambient temperature in Arabidopsis. *Genes Dev*. 21, 397–402. doi: 10.1101/gad.1518407
4. Lei, Y., Lu, L., Liu, H. Y., Li, S., Xing, F., and Chen, L. L. (2014). CRISPR-P: a web tool for synthetic single-guide RNA design of CRISPR-system in plants. *Mol. Plant* 7, 1494–1496. doi: 10.1093/mp/ssu044
5. Michaels, S. D., Himelblau, E., Kim, S. Y., Schomburg, F. M., and Amasino, R. M. (2005). Integration of flowering signals in winter-annual Arabidopsis. *Plant Physiol*. 137, 149–156. doi: 10.1104/pp.104.052811
6. Ongaro, V., Bainbridge, K., Williamson, L., and Leyser, O. (2008). Interactions between axillary branches of Arabidopsis. *Mol. Plant* 1, 388–400. doi: 10.1093/mp/ssn007
7. Rameau, C., Bertheloot, J., Leduc, N., Andrieu, B., Foucher, F., and Sakr, S. (2014). Multiple pathways regulate shoot branching. *Front. Plant Sci*. 5:741. doi: 10.3389/fpls.2014.00741
8. Takada, S., and Goto, K. (2003). Terminal flower2, an Arabidopsis homolog of heterochromatin protein1, counteracts the activation of flowering locus T by constans in the vascular tissues of leaves to regulate flowering time. *Plant Cell* 15, 2856–2865. doi: 10.1105/tpc.016345

Lastly, corrections have been made to the **Methods, Results**, and **Discussion** of the original article and the AGL79KD mutant has been removed as detailed below.

In the **Methods**, subsection **CRISPR-Cas9 Design and Screening for AGL79 Gene Editing** has been removed.

Subsection **Characterization of AGL79 Overexpression Plants** has been corrected to:

“To investigate the role of *AGL79* in Arabidopsis development, we generated transgenic plants with enhanced expression of *AGL79*. Compared to WT, the highest *AGL79* overexpression plants flowered early and had fewer and smaller rosette leaves [Figures 4B, 5B (WT and Group 1)] much like the SPL10 overexpression plants (6mSLP10) (Figure 4C, Supplementary Figure 1). Transgenic Arabidopsis plants harboring the *AGL79* overexpression construct were divided into three groups depending on AGL79 expression. Group 1 (lines L1, L2, and L3) had the highest *AGL79* transcript levels in both the leaf and root tissues (Figure 5A), with lower expression in roots relative to leaves. Group 2 (lines L11, L12, and L20) had intermediate *AGL79* expression, with variable expression levels between leaf and root (Figure 5A). Group 3 (lines L16, L18, and L27) displayed the lowest *AGL79* gene transcripts, and there were no obvious differences in *AGL79* transcript levels between the leaf and root (Figure 5A). Different phenotypes could be observed in these AGL79 overexpression plants depending on AGL79 expression levels (Figure 5B). Compared to WT (3 weeks after seed germination), Group 1 plants displayed fewer rosette leaves and early flowering time (Figure 5B). Group 2 plants displayed a phenotype similar to WT (Figure 5B). Group 3 plants showed more lateral shoot branches and a higher number of rosette leaves, as well as a significant delay in flowering (Figure 5B). In addition, the transcript level of *SPL10* gene was also investigated in both the leaves and roots of the above-mentioned plants. Although changes in *SPL10* expression could be detected in three groups of *AGL79*OE plants (Figure 5C), these changes did not follow any consistent trend, as found for *AGL79* (Figure 5C), suggesting that *AGL79* could be a downstream gene regulated by SPL10, and hence fluctuations in AGL79 expression would not affect the expression of the upstream *SPL10* gene.”

Subsection **Characterization of the AGL79KD Arabidopsis Mutant**, has been removed.

Subsection **Regulatory Relationship between AGL79 and SPL10**, has been corrected to:

“As all the evidence derived from molecular and biological analysis (Figures 2A,B, 4C) revealed that AGL79 is likely regulated through the miR156-SPL pathway, we investigated whether a linear regulatory relationship exists between *SPL10* and *AGL79*. Crossing AGL79OE plants and *spl2spl10* double mutant produced F1 progeny showing WT-like phenotype (Figure 8B). The selected genotyping results of the double mutant (*spl2spl10*) and AGL79 OE plants are shown in Supplementary Figure 3. These results suggest a direct linear relationship between *AGL79* and *SPL10* genes.”

In the **Discussion**, paragraph two has been corrected to:

“The discovery that *AGL79* is regulated by *SPL10* may provide insight into how the latter regulates lateral root development in Arabidopsis (Yu et al., 2015). Currently lateral root formation in Arabidopsis is known to be regulated by two related *AUXIN RESPONSE FACTORS* (*ARF7* and *ARF19*) via direct activation of *LATERAL ORGAN BOUNDARIES DOMAIN* and *ASYMMETRIC LEAVES-LIKE* (*LBD/ASLs*) (Okushima et al., 2007). In addition, lateral root formation in Arabidopsis is also redundantly regulated by cytokinin biosynthesis genes *IPT3* and *IPT5* and all three cytokinin histidine kinase receptor genes (*AHK2, AHK3*, and *CRE1/AHK4*) (Chang et al., 2013). The plant hormones (auxin, cytokinins, gibberellins, abscisic acid, ethylene, jasmonic acid, strigolactones, brassinosteroids, and salicylic acid) also regulate normal root growth and mediate root morphological responses to abiotic stress (Chang et al., 2013). Morphological analysis of Arabidopsis plants with enhanced expression of *AGL79* revealed AGL79 to be involved in controlling shoot branching.”

and paragraph four should be removed and the last paragraph has been corrected to:

“In summary, our results suggest that the miR156/SPL10 regulatory pathway is involved in regulating plant lateral root growth by directly targeting and activating the expression of *AGL79*. By investigating the gain- of function of AGL79 transgenic plants, we also found AGL79 to be involved in regulating plant leaf shape, shoot branching, and flowering time. Further characterization of the *AGL79* gene in other plant species, especially in major crops, will determine how conserved AGL79 is in plants. It can also be tested in crop improvement efforts to enhance resilience and productivity.”

The authors apologize for these errors and state that this does not change the scientific conclusions of the article in any way. The original article has been updated.
