# Supplementary figures and images for: miR156/SPL10 Modulates Lateral Root Development, Branching and Leaf Morphology in Arabidopsis by Silencing AGAMOUS-LIKE 79
Source: Front Plant Sci. 2018 Jan 4;8:2226. doi: 10.3389/fpls.2017.02226 (PMC5758603; doi:10.3389/fpls.2017.02226)

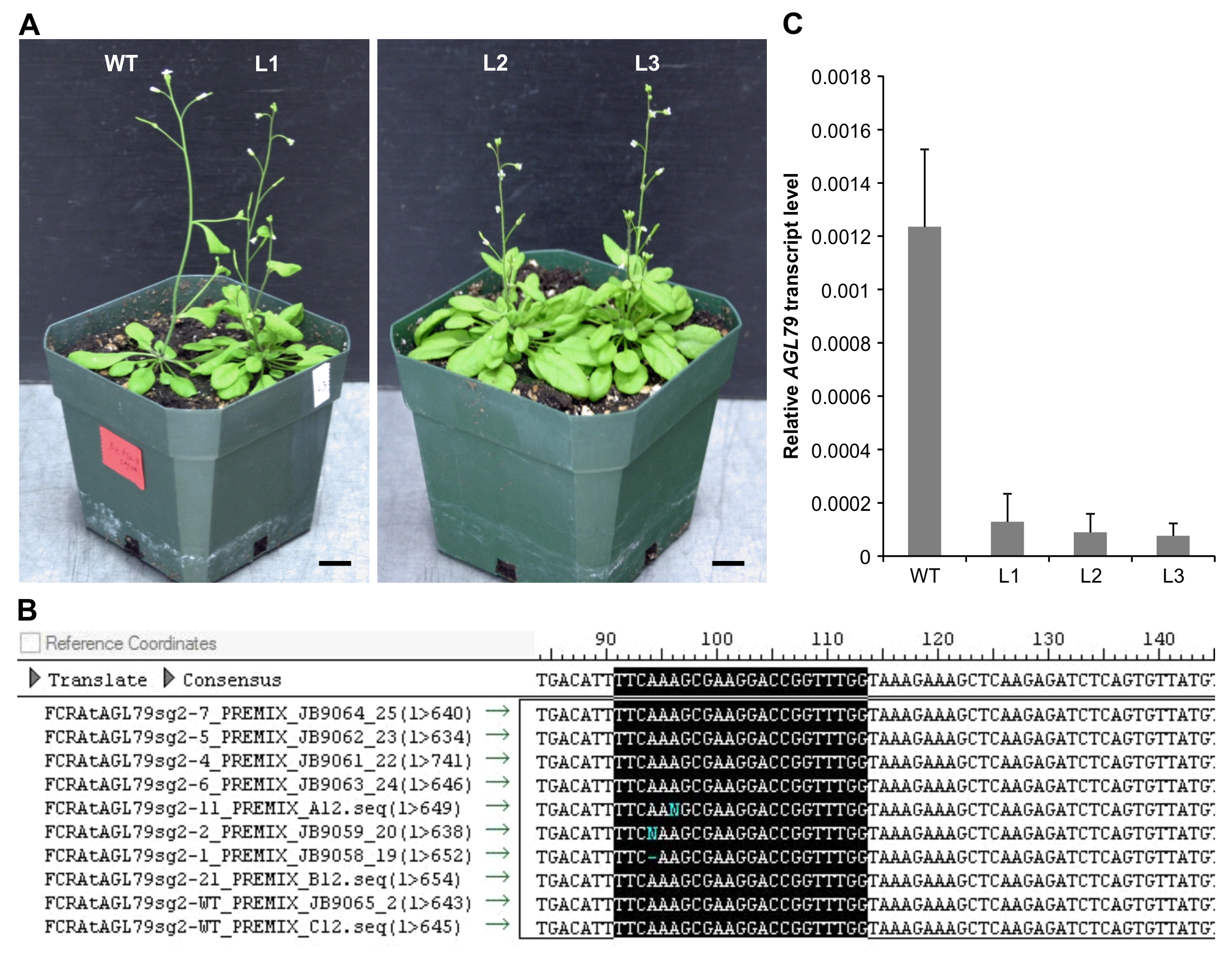

Supplement: Supplementary Figure 1 — AGL79 knockout mutant using CRISPR technique. (A) Vegetative morphology of WT and three lines of AGL79 CRISPR Plants (bar = 1.5 cm). (B) Genomic DNA sequence analysis of CRISPR-modified Arabidopsis plants. (C) Detecting AGL79 transcript levels by qRT-PCR in WT and three lines of AGL79 CRISPR Plants. [file Image1.TIF]

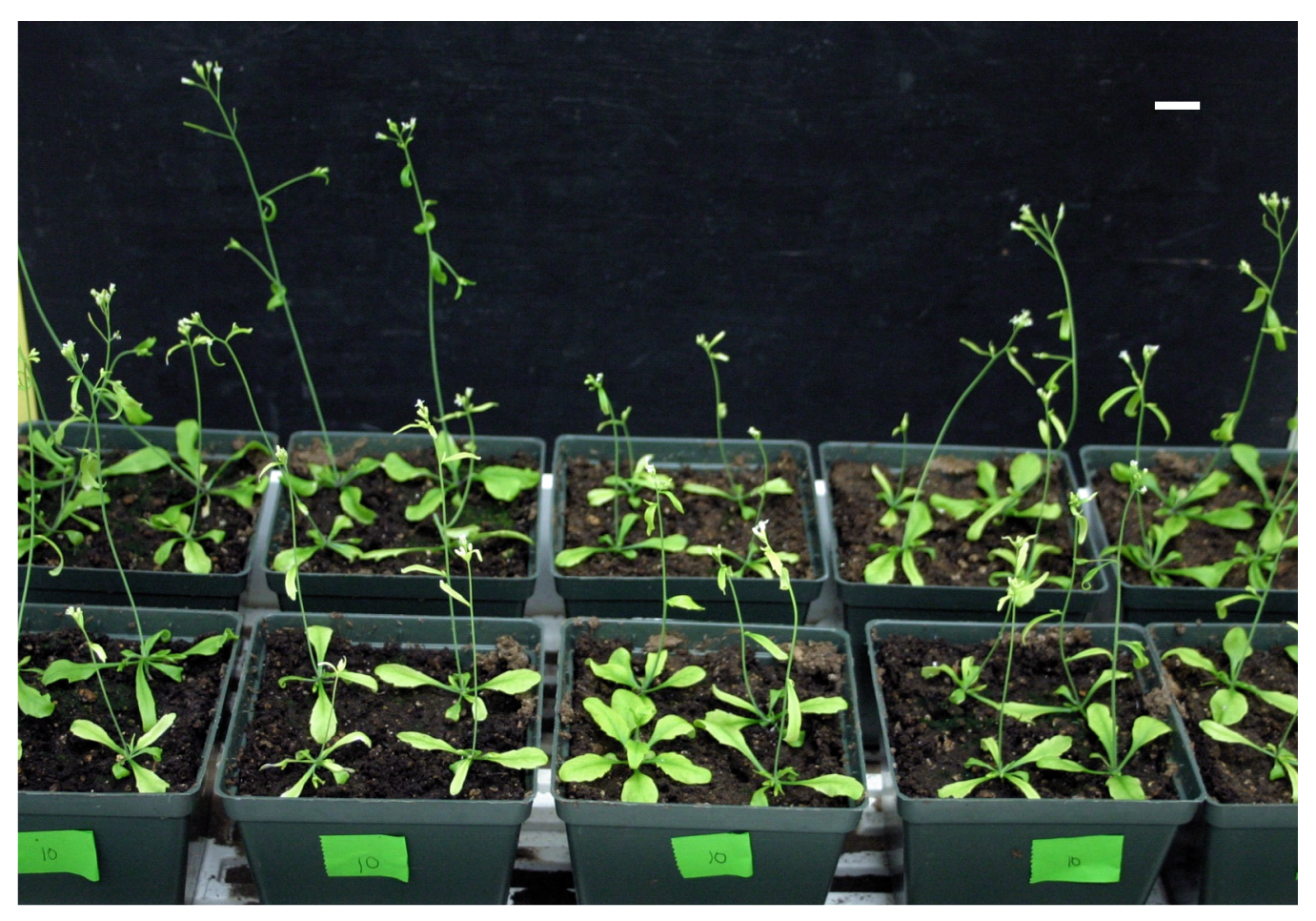

Supplement: Supplementary Figure 2 — Phenotypes of a batch of 6mSPL10 plants (bar = 1.5 cm). [file Image2.TIF]

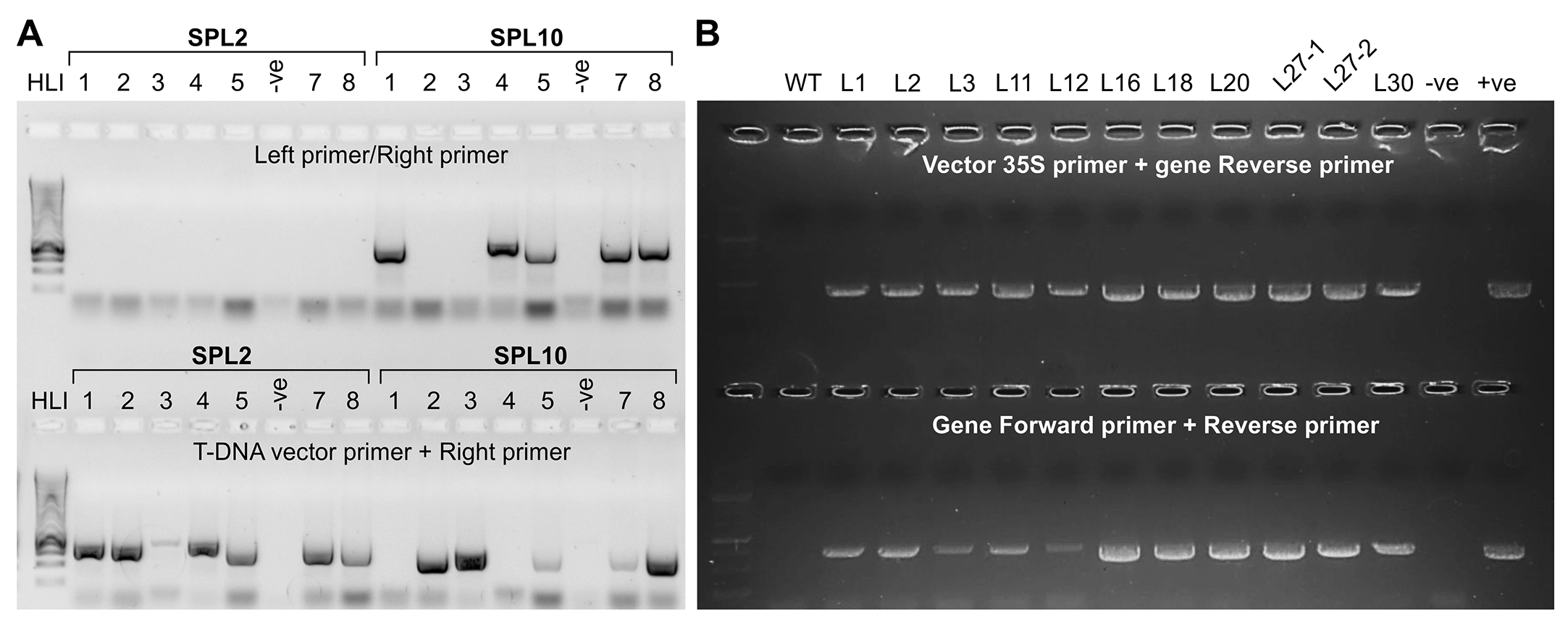

Supplement: Supplementary Figure 3 — Selected genotyping results from plants of (A) spl2spl10 double mutant and (B) AGL79 OE. [file Image3.TIF]
