# Supplementary material for: miR156/SPL10 Modulates Lateral Root Development, Branching and Leaf Morphology in Arabidopsis by Silencing AGAMOUS-LIKE 79
Source: Front Plant Sci. 2018 Jan 4;8:2226. doi: 10.3389/fpls.2017.02226 (PMC5758603; doi:10.3389/fpls.2017.02226)
Supplement: Supplementary Document 1 — The 2,000 bp upstream promoter region of AGL79 gene. [file Presentation1.PDF]

Supplementary Document 1: The 2000 bp upstream promoter region of *AGL79* gene

ATAGAAAAAAGTGGTTATCCAAGCTACACAAACTTTTTAGACTTATTTCTCTCATTCTTC

TAGAGATTTTTAATGAAATTTGTATATACTTGAAATGCATATAAAATAAATAAGAAAACA

ATATCTACTTAATGTCCTGTTCAAGAATTATTAAGAAATTTCACTACGTTGTTAAGTCCA

TTTTACTCCACCTTCTCCACTGGAGTTAGACTCTACTAAGTCGCGTTATCCTGCAGAGTA

AAGATTGACTATGCATTGGACCTTGTCGATGTGAAAATGTTAAAAAGATAATTATTATGA

AATCTCTACTATAACATATTTGGTTTCCTCAATTTTAAAATACCTATTTTTTTCTCAGTT

TACCATTAACCAATCTATTCCGTGATATTTAATGTTTAAACCCTTCTTCAATTCTATTTA

ACCTACATTCTAAACAAGGTGTCCTCATTTTCAAAATCCTAAACTCATCTTCATCTATAT

TTCTATTTTCTGCCAAAATCATACCACAAAAAACATATACGAAAAATGACCACGGAAATT

GTCTTTTATTGTTTATTTCCAAAGTCAACCAAGTTGACTGTTCTTTATATAATGATTTTT

TTTTAAAAAATTATATATTGAGCTAAGAAAATATTAAGTGTAATTTTCAAAAAGAGAAAA

TATATTTATTATAAAAATCTATAGTGGTGGATCAAAATTCTGGTTTAAATTATTGTATCA

ATTTTGAGGTTCAAATTTTAGGATGGAAGAGAAACGTAGAATGCACTTAAAGTTCTGTCG

ATTTTTGTTAGTACATAATCCCTCATATAACCATACATATACTTATTGCAATAACCATAC

GTTAAGAATAGGGACCAACTTCATCGGTGCTTCTGTTAGACATTTCAAGAATGATATATC

ACATTCATGTATCTCCGTATATGTATTAAGACCAATGACTAAACTCAGCCATTGTAGATA

TAGTTCAGGTAAAACTGTCAATTGTGGATCAGTATGTTGAAAATTGTTAACTATGGTTCG

CTTGTAACAGAATATAAATATAATAATAATATATAATACTAATAGTAATATATATATAATAA

TAATAAAAAGCAAGCACTAATTATTTTATAGAAACACTTTAAAAAGTATTAAAATCTCAT

TTAAAACAACTCTCCAAGTTGGTAGTCCATTGATCTACAAGACAGCTCCACCTGAACCAC

ATCCACAATGTAAAATTTAACCGTACACATAAACCATGATCATAAGATGCAACATGGTAA

AATCTCTGGTGAAGTTTTCGTAATCGATGATGAATATATATATATATATATATATATATA

TATATATATATATATATATATATATATATATATATATACAATATATTTTTATTTTCAATC

TATTTAGTAAGTACATATTTGTTATGAAAGTACAAAAATAACATTGTAATAGGTCAAGCT

CTTTGTGGTGGTTTACATTGTAATAGGTCAAGCTCTTTATTATTATTTTACTATATGTAT

CTTCTTATCCATTCTTATATTTTATATCATATATGGTCAAAAGAACCAATCGTATCACAG

CAGCATTTGTTAATAGAACTTTGATCTTGGTAATTACAGGTTCTATATTGAAATCGCAGG

AGCCAAACCAGCAACTTGGGTTAATTATTAACTTCATTCACCAATAATAATAATTGATCG

TCAAGACTATTTCGGACACAAAGACATAAATACTTGTTTTCTGCTCTCCCTTTCCCTAAA

TGTATCATTTGATACTTTCTCTTTTTCCCTCCATATGCCTTTTGTGTGTATAAATATATG

TATATGAATAAGTTCACAAAAATAGAAAATTAAATCAAATCTTCGCGTAATTTCTCATCA

AACTCCACTTTTCTTCTTTTTAAGAGAATCTAGGGTTTCTTCTACTACTTCTTCTTTCTT

CTTTAGGTGGTCAAATTTCCAAGTTCTTCTTTTAGCTCTCTCTTTTGTTTCCAAGAAACC

TGAGAAGAATTTTTGTAGATA

Notes:

The yellow color highlighted sequences are the putative SPL binding sequences (NNGTACR where N=any nucleotide, R=A or G)

The red color sequences are the respective primers that were used to amplify the DNA fragments encompassing the NNGTACR element.
